# Lethal Disease in Dogs Naturally Infected with Severe Fever with Thrombocytopenia Syndrome Virus

**DOI:** 10.3390/v14091963

**Published:** 2022-09-04

**Authors:** Keita Ishijima, Kango Tatemoto, Eunsil Park, Masanobu Kimura, Osamu Fujita, Masakatsu Taira, Yudai Kuroda, Milagros Virhuez Mendoza, Yusuke Inoue, Michiko Harada, Aya Matsuu, Hiroshi Shimoda, Ryusei Kuwata, Shigeru Morikawa, Ken Maeda

**Affiliations:** 1 Department of Veterinary Science, National Institute of Infectious Diseases, 1-23-1 Toyama, Shinjuku-ku, Tokyo 162-8640, Japan; 2 Joint Faculty of Veterinary Medicine, Yamaguchi University, 1677-1 Yoshida, Yamaguchi 753-8515, Japan; 3 Faculty of Veterinary Medicine, Okayama University of Science, 1-3 Ikoinooka, Imabari 794-8555, Japan

**Keywords:** severe fever with thrombocytopenia syndrome virus, dog, zoonosis, RT-PCR, ELISA, animal hospital in Japan

## Abstract

Severe fever with the thrombocytopenia syndrome virus (SFTSV) causes fatal disease in humans, cats, and cheetahs. In this study, the information on seven dogs with SFTS was summarized. All dogs showed anorexia, high fever, leukopenia, and thrombocytopenia, two dogs showed vomiting and loose stool, and five dogs had tick parasites. All dogs also had a history of outdoor activity. The SFTSV gene was detected in all dogs. Remarkably, three dogs (43%) died. SFTSV was isolated from six dogs and the complete genomes were determined. A significant increase in anti-SFTSV-IgG antibodies was observed in two dogs after recovery, and anti-SFTSV-IgM antibodies were detected in four dogs in the acute phase. Using an ELISA cut-off value of 0.410 to discriminate between SFTSV-negative and positive dogs, the detection of anti-SFTSV-IgM antibodies was useful for the diagnosis of dogs with acute-phase SFTS. Four out of the ninety-eight SFTSV-negative dogs possessed high anti-SFTSV IgG antibody titers, indicating that some dogs can recover from SFTSV infection. In conclusion, SFTSV is lethal in some dogs, but many dogs recover from SFTSV infection.

## 1. Introduction

Severe fever with thrombocytopenia syndrome (SFTS) is an emerging infectious disease caused by SFTS virus (SFTSV) infection [1,2]. SFTSV belongs to the order *Bunyavirales*, family *Phenuiviridae*, genus *Bandavirus*, (ICTV, 2019). In December 2012, we isolated the virus from a human SFTS case in Japan [3]. To date, SFTS cases have been reported in China, Korea, Japan, Vietnam and Taiwan [1,3,4,5,6].

SFTSV is one of the arthropod-borne viruses that circulate among ticks and mammals [7]. The major vector tick seems to be *Haemophysalis longicornis*, which maintains the virus across all stages and transmits it transovarially [8]. In Japan, the SFTSV gene has been detected in some species of ticks, including *H. longicornis*, *Amblyomma testudinarium*, and *H. flava* [9]. In Japan, China, and South Korea, the SFTSV gene and anti-SFTSV antibodies have been detected in a number of animal species (e.g., goat, sheep, cattle, deer, elk, pig, wild boar, dog, cat, cheetah, raccoon, mongoose, mink, yellow weasel, chicken, geese, hare, rodent, shrew and hedgehog) [10,11,12,13,14,15,16,17].

In April 2017, we discovered one cat with SFTSV infection in Wakayama Prefecture in Japan. Since then, many cat cases have been reported in the western part of Japan [11]. In addition, cats experimentally inoculated with SFTSV showed severe SFTS and two-thirds of the inoculated cats died [18]. In August 2017, two cheetahs died due to SFTSV infection in Hiroshima Prefecture, Japan [10]. In dogs, SFTSV infection with clinical symptoms has been reported in South Korea [19,20]. These results indicated that some species naturally infected with SFTSV showed clinical symptoms that were similar to those in humans. Furthermore, SFTSV is directly transmitted from diseased cats and dogs to humans [21,22,23,24,25,26]. Thus, SFTSV infection in companion animals is important not only for veterinary medicine, but also for public health.

In this study, we summarize the cases of seven dogs with SFTS in Japan, which occurred after June 2017.

## 2. Materials and Methods

### 2.1. Clinical Samples

Sera or plasma samples and/or oral and rectal swabs of 123 dogs were collected from 91 animal hospitals in 29 prefectures from April 2017 to November 2019. All dogs showed some SFTS-like symptoms, such as fever, lethargy, anorexia, leukopenia, and thrombocytopenia, and veterinarians suspected the possibility of SFTSV infection. Sera and plasma samples were used for RT-PCR, enzyme-linked immunosorbent assay (ELISA) and virus-neutralization (VN) tests and swab samples were used for RT-PCR.

### 2.2. Viral Genome Detection

Viral RNA extraction from serum or plasma and swab samples was performed using a QIAamp Viral RNA Mini Kit (QIAGEN, Hilden, Germany), according to the manufacturer’s protocol. The oral and rectal swabs were suspended in 2 mL of Dulbecco’s Modified Eagle’s medium (DMEM; Thermo Fisher Scientific, Waltham, MA, USA), with 2% fetal calf serum (FCS; Thermo Fisher Scientific) and centrifuged at 2600× *g* for 10 min at 4 °C. One hundred and forty microliters of the supernatants or serum samples were used for RNA extraction. The SFTSV gene was detected by RT-PCR using the QIAGEN OneStep RT-PCR Kit (QIAGEN) and specific primers. Two sets of specific primers were designed from the S segment of SFTSV, which were as follows; SFTSV-S2-200s (5′-GACACAAAGTTCATCATTGTCTTTGCCCT-3′) and SFTSV-S2-360a (5′-TGCTGCAGCACATGTCCAAGTGG-3′), and SFTSV-S7F (5′-GCCATCTGTCTTCTTTTTGCG-3′) and SFTSV-S7R (5′-AGTCACTTGCAAGGCTAAGAGG-3′) [27]. RT-PCR, using SFTSV-S2-200s and SFTSV-S2-360a, was performed as follows: RT reaction at 50 °C for 30 min and at 95 °C for 15 min; 40 cycles of denaturation at 94 °C for 30 s, annealing at 60 °C for 30 s and extension at 72 °C for 1 min; and final extension at 72 °C for 7 min. RT-PCR, using SFTSV-S7F and SFTSV-S7R, was conducted as follows: RT reaction at 50 °C for 30 min and at 95 °C for 15 min; 40 cycles of denaturation at 94 °C for 30 s, annealing at 52 °C for 30 s and extension at 72 °C for 30 s; and final extension at 72 °C for 5 min. The PCR products were confirmed by electrophoresis on 2% agarose and the expected size of fragments amplified with primers SFTSV-S2-200s and SFTSV-S2-360a and SFTSV-S7F and SFTSV-S7R are 201 bp and 125 bp, respectively.

### 2.3. ELISA

Anti-SFTSV IgM and IgG antibodies in dog sera or plasma samples were detected by ELISA. A human hepatoma cell line, HuH-7 cells (Japanese Collection of Research Bioresources [JCRB] number: JCRB0403), was maintained in DMEM containing 10% FCS. After replacement of the medium with DMEM containing 2% FCS, the SFTSV HB-29 strain [1], kindly provided by Dr. Dexin Li and Dr. Mifang Liang at the National Institute for Viral Disease Control and Prevention, Chinese Center for Disease Control and Prevention, was inoculated at a multiplicity of infection of 0.1. For the mock antigen, the same volume of DMEM containing 2% FCS was added. SFTS- and mock-infected HuH-7 were incubated for 3 days at 37 °C in 5% CO_2_. After washing cells with phosphate-buffered saline (PBS), the cells were lysed with 1% Nonidet P-40 (Nacalai Tesque, Kyoto, Japan) in PBS for 5 min. Then, UV exposure was performed to 1 mL lysates in polypropylene tubes (Sarstedt AG & Co. KG, Nümbrecht, Germany) by irradiation with UVB (302 nm) for 10 min. The inactivated lysates were centrifuged at 13,200× *g* for 5 min at 4 °C. The supernatants were collected and stored at −80 °C. One hundred microliters of diluted cell lysates with 0.05 M carbonate-bicarbonate buffer (pH 9.6) at a final concentration of 5 µg/mL were coated onto 96-well microplates (Maxisorp; Nunc, Roskilde, Denmark). After incubation at 37 °C for 2 h, the plates were kept at 4 °C overnight. The antigen was removed, and the wells were incubated with 200 µL per well of 1% Block Ace (Megmilk Snow Brand Co., Ltd., Tokyo, Japan) at 37 °C for 30 min. Wells were washed 3 times with PBS containing 0.05% Tween 20 (FUJIFILM Wako, Osaka, Japan) (PBS-T), and incubated with 100 µL per well of serum or plasma diluted to 1:100 with PBS-T containing 0.4% Block Ace, at 37 °C for 30 min. Antibodies against canine IgM and IgG conjugated with horseradish peroxidase (HRP) (Bethyl Laboratories, Montgomery, TX, USA) were diluted with PBS-T containing 0.4% Block Ace. After being washed 3 times with PBS-T, 100 µL per well of the diluted second antibodies was added and the plate was incubated at 37 °C for 30 min. Following the 3 washes with PBS-T, 100 µL of substrate reagent (ABTS Microwell Peroxidase Substrate; SeraCare Life Sciences, Inc., Milford, MA, USA) was added to each well. After shaking at room temperature for 30 min, the reaction was stopped by adding 100 µL of 1% sodium dodecyl sulfate to each well. Absorbance was measured using a spectrophotometer (Bio-Rad, Hercules, CA, USA) at a wavelength of 405 nm. All optical density (OD) values in the mock-infected cell lysates were subtracted from those in the SFTS-infected cell lysates.

### 2.4. Virus Isolation

SFTSV was isolated from sera and plasma samples of RT-PCR-positive dogs. Serum or plasma was inoculated into Vero cells (JCRB9013) and the cells were incubated at 37 °C under 5% CO_2_. After observation of the cytopathic effect (CPE), the culture supernatants were collected. Supernatants collected after centrifugation at 2600× *g* for 10 min were stored at −80 °C. RNA for viral genome amplification was extracted from the supernatants using the QIAamp Viral RNA Mini Kit (QIAGEN).

### 2.5. Viral Genome Amplification and Genome Sequencing

For the determination of the complete viral genome sequence, RT-PCR was performed as described previously [28], with minor modifications. A QIAGEN OneStep RT-PCR Kit (QIAGEN) was used. The PCR products were analyzed by direct sequencing. The nucleotide sequences of SFTSV were deposited in the DNA Data Bank of Japan (DDBJ) databases (Accession numbers: Ydog1 (LC570802), Ydog34 (LC570784, LC570785, and LC570786), Ydog39 (LC570787, LC570788, and LC570789), Ydog59 (LC570790, LC570791, and LC570792), Ydog63 (LC570793, LC570794, and LC570795), Ydog70 (LC570796, LC570797, and LC570798), and N100 (LC570799, LC570800, and LC570801)).

### 2.6. Phylogenetic Analysis of SFTSV

The sequences of SFTSV, including sequences of the Guertu virus [29] as an outgroup, were output as three FASTA files (S, M, and L segments). Sequence alignments were performed using the MUSCLE software program, supported by the MEGA 10.0 software program [30]. The optimal DNA model was determined for each segment, and a phylogenetic analysis was performed by the maximum likelihood method using the MEGA 10.0 software program. The statistical significance of the resulting trees was evaluated by a bootstrap test with 1000 replications.

### 2.7. Fifty Percent Focus Reduction Neutralization Titer (FRNT_50_)

FRNT_50_ was determined as described previously [18], with a minor modification. Approximately 300 focus-forming units of SFTSV HB-29 [1] were mixed with an equal volume of serially-diluted heat-inactivated sera and incubated for 1 h at 37 °C. One hundred microliters of mixture was inoculated into confluent monolayers of Vero cells in 12-well plates and incubated for 1 hour at 37 °C. The inocula were removed, and the cells were washed once with DMEM containing 2% FCS. Plates were incubated at 37 °C in 5% CO_2_ in DMEM containing 2% FCS and 0.8% methylcellulose for 1 week. Cells were fixed with 10% buffered formalin and exposed to UV light to inactivate virus for more than 30 min. The cells were permeabilized with 0.1% Triton X-100 (Merk, Darmstadt, Germany) and stained with rabbit antibodies against SFTSV-NP [31] as a primary antibody and HRP-conjugated recombinant protein A/G (Thermo Fisher Scientific) as a secondary antibody. The plaques were visualized with 3, 3′-diaminobenzidine tetrahydrochloride (peroxidase stain DAB kit (brown stain), Nacalai Tesque, Japan). The values of FRNT_50_ were determined as a reciprocal of the highest dilution when the number of the plaques was <50% of the number of plaques in wells without serum.

### 2.8. Receiver Operating Characteristic (ROC) Analysis

We performed an ROC analysis to determine the optimum cut-off value of the anti-SFTSV IgM ELISA in dogs. The sensitivity and specificity were estimated according to the results of SFTSV gene detection as the gold standard. The ROC analysis was performed using the GraphPad Prism software program (version 5.0, GraphPad, Inc., San Diego, CA, USA).

## 3. Results

### 3.1. First Diagnosis of SFTS in Dogs

In June 2017, one veterinarian in Tokushima Prefecture asked us to test a patient’s dog for SFTSV because the clinical symptoms, including fever, diarrhea, leukopenia and thrombocytopenia, were similar to SFTS in humans [26]. The data of the dog are included in the present report as Dog No.1. Just two months prior, we had diagnosed the first cat with SFTS in Wakayama Prefecture. We conducted RT-PCR and ELISA and found that the SFTSV gene and anti-SFTSV IgM antibody were detected in the dog serum [26]. Unfortunately, we could not isolate SFTSV from the dog. Since then, we have observed a total of seven dogs with SFTS up until November 2019. All of the dogs were confirmed to be infected with SFTSV by RT-PCR.

### 3.2. Information on the Dogs with SFTS

The clinical characteristics of the seven dogs (uncastrated male, *n* = 3; castrated male, *n* = 1; spayed female, *n* = 3) with SFTS are shown in Table 1. The ages of the seven dogs with SFTS ranged from 3 to 13 years. In particular, five dogs with SFTS were 3–5 years of age. Concerning the breeds of the seven dogs, four were mongrels, one was a Poodle, and one was a Welsh Corgi. All had a history of outdoor activities. Two dogs with SFTS were treated with tick medication within 1 month prior to the onset.

### 3.3. Clinical Symptoms of SFTS Dogs

The following symptoms were observed in all the seven dogs: decreased activity, fever (≥39 °C), leukopenia (<6000 /µL), and thrombocytopenia (<200,000/µL). One dog had diarrhea and bloody stool. Vomiting was observed in two dogs and one also had diarrhea. However, the other five dogs had no gastrointestinal symptoms. Five of the seven dogs with SFTS were bitten by ticks at the onset of SFTS or at visiting animal hospitals. One dog was bitten by ticks 2 days before the onset. Three of the seven dogs with SFTS died within 5 days after the time of symptom onset. Four dogs with SFTS recovered, and serum could be collected sequentially from two of the recovered dogs.

### 3.4. Laboratory Findings in SFTS Dogs

The laboratory findings of the dogs with SFTS upon their first visit to an animal hospital are shown in Table 2. High alanine aminotransferase (ALT) was observed in six of the seven dogs, and high aspartate transaminase (AST) was found in three dogs. High total bilirubin was observed in only one of the two examined dogs. High C-reactive protein (CRP) was observed in all of the three dogs.

### 3.5. Detection of Anti-SFTSV Antibodies by ELISA

**Figure 1:**
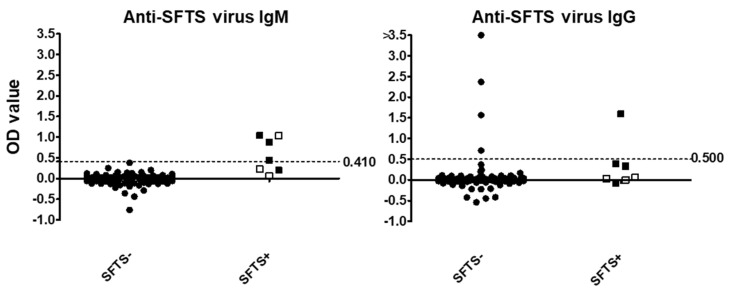
Detection of anti-SFTSV antibodies in dog sera. OD values of the ELISA are plotted. Broken lines indicate the cut-off value. Left figure shows the plot of the OD values of the IgM ELISA, while the right figure shows the plot of the OD values of the IgG ELISA. Dogs were divided into two groups, SFTSV + or −, according to the results of RT-PCR for SFTSV. Open boxes indicate dogs that died due to SFTS infection.

For the serological diagnosis of SFTSV infection in dogs, IgM and IgG ELISA was performed using 116 and 105 serum samples, respectively (Figure 1). The results showed that in dogs with and without SFTS, the median OD value of anti-SFTSV IgM was 0.440 and 0.000, respectively. Based on an ROC analysis of the OD value of anti-SFTSV IgM, the cut-off value that showed the highest specificity was 0.410. Finally, four dogs were diagnosed as positive by the ELISA for anti-SFTSV IgM antibodies. Importantly, two of the three dogs that died due to SFTSV infection were negative for anti-SFTSV IgM antibodies (white circles in Figure 1). On the other hand, three of the four dogs that recovered possessed anti-SFTSV IgM antibodies during their first visit. In the convalescent phase, the anti-SFTSV IgM antibody titers of the two recovered dogs were below the cut-off value (Table 3).

In this study, the cut-off value of IgG was tentatively set at 0.500, because we could not determine the cut-off value of IgG. Now, we are performing research to determine the cut-off value of IgG in dogs by comparison between the VN test and ELISA. At the time, anti-SFTSV IgG antibodies were detected in one dog with SFTS during the first visit and from all recovered dogs during the convalescent phase (Table 3). On the other hand, anti-SFTSV IgG antibodies were detected in four dogs without SFTS, suggesting that these dogs had been previously infected with SFTSV and recovered.

### 3.6. Detection of VN Antibodies

**Figure 2:**
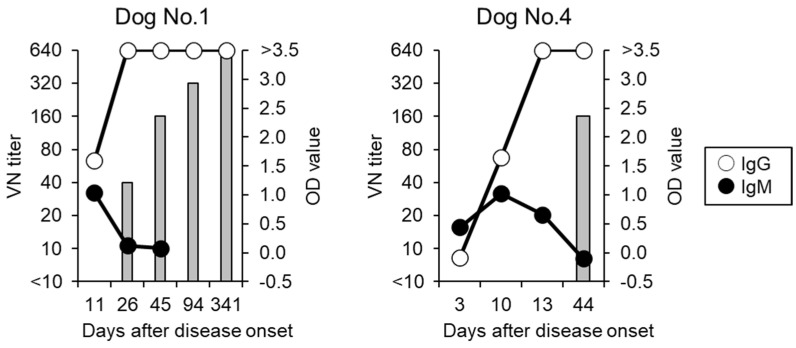
Changes in antibody titers of dogs with SFTS during observation. Lines indicate the OD values of the ELISA. Open and closed circles show IgG and IgM antibodies, respectively. Gray bars show VN titer by FRNT_50_.

At the onset of SFTS, the VN titer was below the limit of detection (<1:10) in all dogs (Table 3). Sera were collected continuously for two of the four dogs that recovered (Dog No.1 and 4). An increased VN titer was detected at 26 days post-infection (d.p.i.) in Dog No.1 and 14 d.p.i. in Dog No.4. The VN titer of Dog No.1 continued to increase, and reached 1:640 at 341 d.p.i. (Table 3 and Figure 2).

The VN titers of four dogs without SFTS that possessed anti-SFTSV IgG antibodies were measured, revealing that the titers were ≥1:80 in three dogs, and <1:10 in one dog.

### 3.7. Virus Isolation and Their Characterization

**Figure 3:**
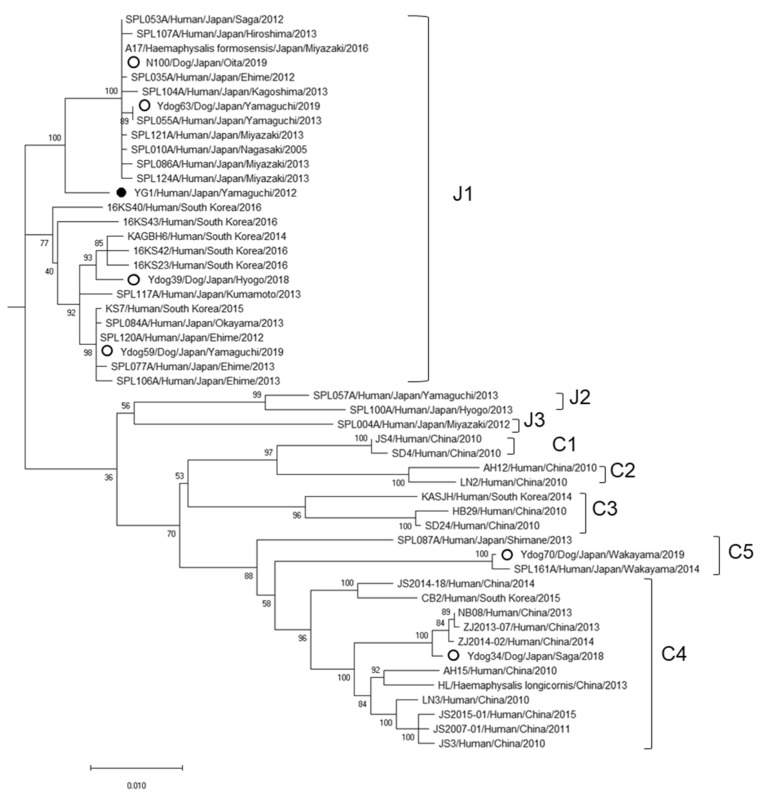
Phylogenetic analysis by the maximum likelihood method based on the nucleotide sequences of small (S) segments (1697 bp). The Guertu virus used as an outgroup is not shown. The open circles indicate the virus strain isolated from a dog in this study. The closed circle indicates the virus strain isolated from the first human patient in Japan. The genotype is shown to the right of the strain name 3.

Virus isolation was performed using Vero cells and six isolates were obtained (Table 3). Their almost complete genomes were determined and compared with previously reported SFTSV strains. Each strain was classified as follows, according to the previously reported genotypes [28]: J1, four strains; C4, one strain; and for C5, one strain (Figure 3). Ydog63, one of the four isolates classified as J1, showed the highest homology in the S and L segments with the human-derived SFTSV strain isolated in the same prefecture. On the other hand, Ydog59 and N100 showed the highest homology with isolates from different prefectures in Japan, and Ydog39 showed the highest homology with Korean isolates. Ydog34, the one isolate classified as C4, showed the highest homology with isolates in South Korea and China. Ydog70, the one isolate classified as C5, showed the highest homology with an SFTSV strain isolated from a cat and a human in the same prefecture (Supplementary Table S1).

## 4. Discussion

Seven dogs with SFTS were found during 2017–2019 in this study. While a few studies on cats with clinical symptoms of SFTS have been reported in Japan [11,18], this is the first report to collect and compare the clinical information of multiple dogs with SFTS. This is also the first report of lethal SFTSV infection in dogs. Interestingly, the number of dogs with SFTS is apparently smaller in comparison to the number of cats with SFTS. Matsuu et al. reported that SFTSV infection was detected in 19.5% of 123 cats in which SFTS was suspected by a veterinarian [11]. In our study during 2017–2020, SFTSV infection was detected in 21.1% of 494 cats in which SFTS was suspected by a veterinarian (manuscript in preparation). On the other hand, 5.7% (7/123) of the dogs with suspected SFTS were finally diagnosed with SFTS in this study. The results suggest that the symptoms of SFTSV infection in dogs may not be as clear as those in cats. In addition, anti-SFTSV IgG antibodies were detected in some dogs without SFTS in this study (Figure 1). These results suggest that many dogs infected with SFTSV may be asymptomatic or mild. To elucidate the pathogenicity of SFTSV for dogs, studies that include approaches such as experimental infection in dogs are required.

The ELISA for detecting canine IgM against SFTSV was established by the comparison between the dogs with and without SFTS (Figure 1). All dogs without SFTS were negative for IgM against SFTSV in this ELISA, but some dogs with SFTS were positive. Therefore, the IgM ELISA may assist in the diagnosis of SFTS, and IgM-positive dogs can be diagnosed as dogs with acute SFTSV infection. On the other hand, IgM-negative dogs cannot deny SFTSV infection. Two dogs that recovered from SFTS had IgM elevation in the acute phase and decay in the recovery phase (Figure 2). The detection of IgM against SFTSV proved to be an effective method for diagnosing dogs with acute-phase SFTS. Similarly, two dogs that recovered from SFTS showed IgG elevation in the recovery phase (Figure 2). These results confirmed that the detection of IgG in paired serum samples is effective for the diagnosis of dogs with SFTS. These results indicated that IgG ELISA can be used in the serological surveillance of SFTSV infection, because the anti-SFTSV IgG antibody was maintained for longer than the anti-IgM antibody in these recovered dogs. In addition, none of the dogs with SFTS possessed VN antibodies against SFTSV in the acute phase, but VN antibodies increased in the recovery phase. Interestingly, the VN titers kept increasing up to 341 days after the onset of disease in one dog. The virus antigen that remained in the dog may continuously stimulate the immune system. Further studies will be required to clarify persistent infection with SFTSV.

The clinical presentations of dogs with SFTS were similar to those of cats [11,18,32,33], cheetahs [10], and human patients [34] with SFTS. Although dogs with SFTS presented with fever, leukocytopenia and thrombocytopenia, these are universal symptoms in dogs with a wide range of viral infections, and are not specific to SFTS. CRP was one of the differences between human patients and dogs with SFTS. High CRP values were observed in dogs with SFTS, but not human patients with SFTS [34]. This may result from coinfection with other pathogens; hence, it is difficult to state whether high CRP is a specific presentation in dogs with SFTS. High serum amyloid A (SAA) concentrations were frequently found in cats with SFTS [11]. SAA is a marker of inflammation and infection in cats, as CRP is in dogs. It was suggested that SFTSV infection in cats and dogs induces inflammation more strongly than it does in humans.

In the present study, all dogs with SFTS received symptomatic therapy, as there is currently no SFTS-specific treatment. In the recovered dogs, VN antibodies were detected in the sera collected more than 3 weeks after the onset of SFTS. In contrast, ELISA revealed that IgG against SFTSV was elevated in sera collected 10 or 11 days after the onset (Figure 2). Suzuki et al. reported that SFTSV predominantly infected B cell-lineage lymphocytes [35]. As with SFTS patients, infection of B cell-lineage lymphocytes by SFTSV may delay the induction of VN antibodies in dogs with SFTS.

The mortality rate of dogs with SFTS was 43%. This was lower than the mortality rate of 70% that has been reported in cats [11]. The result might be attributed to the low number of cases of dogs with SFTS. Meanwhile, we have concluded that SFTS is a noteworthy lethal viral disease of dogs in Japan, which is free of rabies and where canine distemper and canine parvovirus are controlled by vaccines.

In the present study, other than blood samples, the SFTSV gene was detected in fecal swabs (Dog No.2 and No.7) and an oral swab (Dog No.7). This result was similar to the results observed in cats with SFTS [11,18]. Several cases of cat-to-human SFTSV infection have been reported in Japan [22,23,25]. Kirino et al. [36] reported that the rate of SFTSV seropositivity in small-animal veterinarians and nurses was significantly higher in comparison to healthy blood donors. In addition, the owner of Dog No.1 showed the onset of symptoms of SFTS at almost the same time as Dog No.1. Dog-to-human infection was suspected in that case [26]. In addition to symptoms and treatments for dogs with SFTS, we need to raise awareness of the countermeasures to prevent SFTSV infection in veterinarians, veterinary nurses and pet owners.

Two strains of SFTSV isolated in this study were closely related to the strains isolated in China and Korea (Figure 3, genotype C4 and C5). Although SFTS is an emerging infectious disease, the origin is still unknown. One hypothesis is that migratory birds might have carried the ticks infected with SFTSV from the Eurasian continent [28,37]. We are currently analyzing the viruses in biting ticks collected from migratory birds.

## 5. Conclusions

Information about the diagnosis of SFTS in dogs in Japan was summarized in this study. We hope that the information will be useful for the clinical diagnosis of SFTS in dogs. The established ELISA can be used for surveillance, as well as the diagnosis of SFTS. The transmission of SFTSV from companion animals, including dogs, to humans has been reported. These reports have demonstrated that SFTS is crucial not only as a meta-zoonosis, but also as a direct zoonosis. The rapid diagnosis of SFTS in companion animals would reduce the risk of SFTSV infection in humans. Finally, since SFTSV causes lethal disease in the dog population, preventive methods, including a vaccine, should be developed as soon as possible.

## Figures and Tables

**Table 1:** Information on the dogs with SFTS (*n* = 7).

Characteristics	Number (%)
**Sex**	
Uncastrated male	3 (43%)
Castrated male	1 (14%)
Spayed female	3 (43%)
**Age**	
3–5 years old	5 (71%)
>10 years old	2 (29%)
**Breed**	
Mongrel	4 (57%)
Poodle (Toy)	1 (14%)
Welsh Corgi	1 (14%)
No record	1 (14%)
**Environment**	
Indoor and outdoor	3 (43%)
Mainly outdoor	4 (57%)
**Tick medication**	
Within 1 month before onset	2 (29%)
No medication	2 (29%)
No record	3 (43%)

**Table 2:** Laboratory findings of the dogs with SFTS at the first visit.

	Unit	Number ^4^	Median	95% Confidence Interval
Body temperature	°C	7	40.7	40.1 to 41.1
RBC	×10^4^/µL	7	718	600 to 844
WBC	/µL	7	2400	1736 to 4350
Platelet	/µL	7	65,000	7071 to 118,072
ALT ^1^	IU/L	6	124	0 to 662
AST	IU/L	3	677	0 to 1387
CRP ^2^	mg/dL	3	7	7 to 7
LIP	IU/L	2	185	0 to 2040
CPK	IU/L	2	127.5	0 to 769
ALP	IU/L	2	528.5	0 to 2542
T-bil ^3^	mg/dL	2	0.8	0 to 7.2

^1^ The upper limit of ALT was tentatively set to 1000 IU/L. ^2^ The upper limit of CRP was tentatively set to 7.0 mg/dL. ^3^ The lower limit of T-bil was tentatively set to 0.3 mg/dL. ^4^ The number indicates number of dogs with results of examination.

**Table 3:** Changes in antibody titers in dogs with SFTS.

	Date of the First Hospitalization (y/m/d)	Virus Isolation (Strain)	Outcome			Change in Antibody				
1 *	2017/6/3	-	Recovered	Days after onset		11	26	45	94	341
				VN titer		<1:10	**1:40**	**1:160**	**1:320**	**1:40**
				ELISA	IgM	**1.04**	0.124	0.08	N.D.	N.D.
					IgG	**1.595**	**>3.5**	**>3.5**	**>3.5**	**>3.5**
2	2018/6/3	+ (Ydog34)	Recovered	Days after onset		4				
				VN titer		<1:10				
				ELISA	IgM	**0.88**				
					IgG	0.03				
3	2018/8/28	+ (Ydog39)	Dead	Days after onset		2				
				VN titer		<1:10				
				ELISA	IgM	**1.03**				
					IgG	0.03				
4	2019/4/2	+ (Ydog59)	Recovered	Days after onset		3	10	13	44	
				VN titer		<1:10	<1:10	<1:10	**1:160**	
				ELISA	IgM	**0.44**	**1.02**	**0.66**	−0.10	
					IgG	−0.08	**1.65**	**>3.5**	**>3.5**	
5	2019/4/25	+ (Ydog63)	Recovered	Days after onset		1				
				VN titer		<1:10				
				ELISA	IgM	0.20				
					IgG	0.39				
6	2019/5/16	+ (Ydog70)	Dead	Days after onset		1				
				VN titer		<1:10				
				ELISA	IgM	0.06				
					IgG	0.00				
7	2019/6/7	+ (N100)	Dead	Days after onset		4				
				VN titer						
				ELISA	IgM	0.22				
					IgG	0.06				

* Data of this dog were reported previously [26]. Bold letters indicate positive sample.

## Data Availability

Not applicable.

## Supplementary Materials

The following supporting information can be downloaded at: https://www.mdpi.com/article/10.3390/v14091963/s1, Table S1: SFTSV strains to which isolates in this study showed highest homology.
