# Supplementary material for: Lethal Disease in Dogs Naturally Infected with Severe Fever with Thrombocytopenia Syndrome Virus
Source: Viruses. 2022 Sep 4;14(9):1963. doi: 10.3390/v14091963 (PMC9502089; doi:10.3390/v14091963)
Supplement: Supplementary file 1 [file viruses-14-01963-s001.zip › viruses-1885858-supplementary.pdf]

Supplementary Table S1. SFTSV strains to which isolates in this study showed highest homology

Strains	Prefecture	Segment	DDBJ accession number	Most closely related strains	The highest identities	Country (Prefecture)	Host
Ydog34	Saga	S	LC570784.1	KAGWH2	1692/1697 (99.71%)	South Korea	Human
		M	LC570785.1	KAICH	3318/3333 (99.55%)	South Korea	Human
		L	LC570786.1	ZJ2014-02	6313/6324 (99.83%)	China	Human
		All		ZJ2014-02	11316/11354 (99.67%)	China	Human
Ydog39	Hyogo	S	LC570787.1	DP01-Korea-13	1689/1697 (99.53%)	South Korea	Human
		M	LC570788.1	KAGBH	3305/3333 (99.16%)	South Korea	Human
		L	LC570789.1	16KS42	6292/6324 (99.49%)	South Korea	Human
		All		16KS42	11280/11354 (99.35%)	South Korea	Human
Ydog59	Yamaguchi	S	LC570790.1	SPL120A	1697/1697 (100%)	Japan (Ehime)	Human
		M	LC570791.1	KS7	3325/3333 (99.76%)	South Korea	Human
		L	LC570792.1	SPL120A	6314/6324 (99.84%)	Japan (Ehime)	Human
		All		SPL120A	11333/11354 (99.82%)	Japan (Ehime)	Human
Ydog63	Yamaguchi	S	LC570793.1	SPL055A	1697/1697 (100%)	Japan (Yamaguchi)	Human
		M	LC570794.1	SPL075A	3330/3333 (99.91%)	Japan (Kochi)	Human
		L	LC570795.1	SPL055A	6318/6324 (99.91%)	Japan (Yamaguchi)	Human
		All		SPL055A	11344/11354 (99.91%)	Japan (Yamaguchi)	Human
Ydog70	Wakayama	S	LC570796.1	SPL161A	1693/1697 (99.76%)	Japan (Wakayama)	Human
		M	LC570797.1	KUVL-87	3290/3317 (99.19%)	Japan (Wakayama)	Cat
		L	LC570798.1	SPL161A	6298/6324 (99.59%)	Japan (Wakayama)	Human
		All		JS-2014-18	10945/11354 (96.40%)	China	Human
N100	Oita	S	LC570799.1	A17, SPL053A	1697/1697 (100%)	Japan	Cat and human
		M	LC570800.1	SPL075A	3323/3333 (99.70%)	Japan (Kochi)	Human
		L	LC570801.1	SPL124A	6310/6324 (99.78%)	Japan (Miyazaki)	Human
		All		SPL124A	11326/11354 (99.75%)	Japan (Miyazaki)	Human
